# ProSight Native: Defining Protein Complex Composition from Native Top-Down Mass Spectrometry Data

**DOI:** 10.1021/acs.jproteome.3c00171

**Published:** 2023-07-12

**Authors:** Kenneth R. Durbin, Matthew T. Robey, Lilien N. Voong, Ryan T. Fellers, Corinne A. Lutomski, Tarick J. El-Baba, Carol V. Robinson, Neil L. Kelleher

**Affiliations:** † Proteinaceous, Inc., Evanston, Illinois 60201, United States; ‡ Northwestern University, Evanston, Illinois 60208, United States; § Department of Chemistry, University of Oxford, 12 Mansfield Rd. Oxford OX1 3TA, U.K.; ∥ Kavli Institute for NanoScience Discovery, Dorothy Crowfoot Hodgkin Building University of Oxford, Oxford OX1 3QU, U.K.

**Keywords:** top down mass spectrometry, native mass spectrometry, protein complex, intact protein, bioinformatics, protein search engine, deconvolution

## Abstract

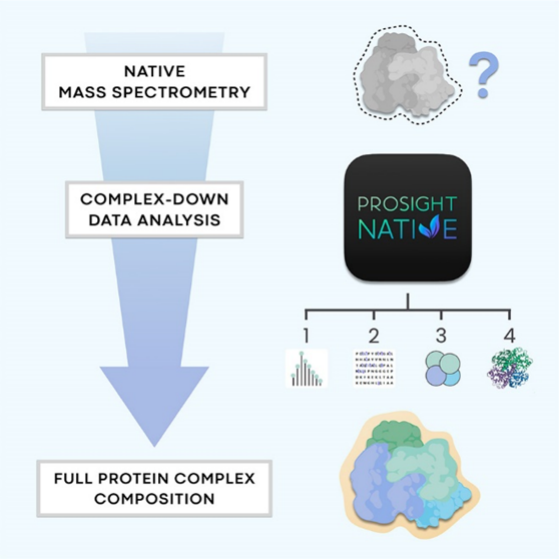

Native mass spectrometry has recently moved alongside traditional structural biology techniques in its ability to provide clear insights into the composition of protein complexes. However, to date, limited software tools are available for the comprehensive analysis of native mass spectrometry data on protein complexes, particularly for experiments aimed at elucidating the composition of an intact protein complex. Here, we introduce ProSight Native as a start-to-finish informatics platform for analyzing native protein and protein complex data. Combining mass determination via spectral deconvolution with a top-down database search and stoichiometry calculations, ProSight Native can determine the complete composition of protein complexes. To demonstrate its features, we used ProSight Native to successfully determine the composition of the homotetrameric membrane complex Aquaporin Z. We also revisited previously published spectra and were able to decipher the composition of a heterodimer complex bound with two noncovalently associated ligands. In addition to determining complex composition, we developed new tools in the software for validating native mass spectrometry fragment ions and mapping top-down fragmentation data onto three-dimensional protein structures. Taken together, ProSight Native will reduce the informatics burden on the growing field of native mass spectrometry, enabling the technology to further its reach.

## Introduction

Proteins drive nearly every cellular process in biology. By forming complexes with each other, proteins expand their functional repertoire to carry out distinct biological activities as new molecular machinery. Thus, defining the composition of protein complexes, as well as understanding their compositional dynamics, offers important insights into the molecular mechanisms that govern development and disease.^1^

Mass spectrometry has emerged as a powerful tool for investigating protein complexes and is used to complement conventional biophysical techniques such as X-ray crystallography, NMR, and electron microscopy.^2^ Over the past decade, improvements in mass spectrometry technologies have forged new avenues for characterizing intact protein complexes in their native state. Accordingly, native mass spectrometry (nMS) has become important for structural investigations of protein assembly, stoichiometry, and three-dimensional architecture.^3^

Protein complexes can now be completely characterized in a single nMS experiment by combining intact complex analysis with the full sequencing of proteoform subunits. This technique, coined “complex-down MS”, uses nondenaturing conditions and multistage tandem MS to elucidate complexes in the gas phase.^4^ Complex-down analysis has three main components: (1) complexes are analyzed in their intact native state, (2) constituent subunits are ejected from the complex, and (3) each subunit is fragmented for protein identification. All three components can then be combined to reconstruct the protein complex composition. What was once a difficult experiment can now be routinely accomplished with modern mass spectrometers.

Despite advances in instrument and sample preparation, one of the most significant barriers of successfully performing complex-down experiments is data analysis. Presently, no software platform exists that covers all three major components of complex-down analysis: intact mass determination, subunit identification, and stoichiometry inferences. Over the last several decades, many software tools have been created for deconvolving mass spectra to obtain protein masses. Among the most popular for nMS experiments is UniDec,^5^ which takes a Bayesian approach to find the most probable mass species from an *m*/*z* spectrum. Other commonly used algorithms include the parsimonious Intact Mass deconvolution algorithm as well as the ReSpect and Xtract algorithms that drive the sliding window deconvolution in BioPharma Finder (Thermo Fisher Scientific).^6,7^ While these tools produce robust deconvolution output, they have not been directly paired with both downstream mass identification and stoichiometry calculations. To circumvent these gaps in nMS data analysis, results must be patched together using multiple software tools. Currently, mass identification can be accomplished in one of three ways. First, masses can be matched to candidates via an “intact mass tag” search (e.g., the Intact workflow of BioPharma Finder).^8^ Second, proteoform sequences can be manually matched to fragmentation spectra or fragment ions using tools such as LcMS-Spectator, ProSight Lite, or TDValidator.^9–11^ Third, an automated top-down proteomics search can be conducted to obtain subunit proteoform identification. Common top-down search engines are ProSightPD and TopPIC.^12,13^ Following proteoform identification, the stoichiometry of the protein complex is usually manually calculated. While early software tools have automated stoichiometry calculation,^14^ they still require other software to define the input data.

Although combining complementary informatics tools for complex-down MS can yield successful results, bridging the data across them can be tedious and requires significant knowledge and input. These challenges present a substantial barrier of entry to using nMS for structural biology applications. Here, we introduce ProSight Native, a novel software suite that simplifies and reduces to practice the informatics of native and intact protein mass spectrometry. While the ProSight Native platform can handle a wide range of intact and top-down MS workflows, including high-throughput protein deconvolution, here, we focus on the complex-down workflow. ProSight Native features built-in algorithms that can perform every analysis step necessary for determining the full composition of a protein complex. These steps include the following: (1) determining the mass of the intact complex and associated subunits through protein deconvolution, (2) characterizing proteoform subunits through top-down database search, and (3) inferring the stoichiometry by considering subunit combinations that constitute the mass of the intact complex. Altogether, ProSight Native is an end-to-end software solution for unveiling the complete composition of protein complexes.

## Methods

### ProSight Native

The ProSight Native software platform is a desktop application built in the .NET Windows Presentation Framework. General descriptions of the software are included in the main text. Overall, the default settings for the THRASH, kDecon, and search portions of ProSight Native were used, except for the maximum mass parameter of the deconvolution algorithms, which was set to be greater than the masses present in the spectra being analyzed. The signal-to-noise was calculated using the sampled noise values from the Thermo .raw files. For search database input, .xml files containing the sequence and known UniProt modifications were used. These .xml files were made with the freeware tool, ProSight Annotator.^15^

### Sample Preparation and Data Collection

Aquaporin Z (AqpZ) was expressed in *Escherichia coli* BL21 (DE3) cells and purified as previously described.^16^ AqpZ was buffer exchanged into 200 mM ammonium acetate containing 2× the critical micelle concentration of tetraethylene glycol monooctyl ether (C8E4) detergent using size exclusion chromatography with a Superdex 200 Increase column (Cytiva, Marlborough, MA). AqpZ was concentrated to ∼12 μM, aliquoted, flash frozen in liquid nitrogen, and stored at −80 C. nMS was performed on a Thermo Q Exactive Ultra High Mass Resolution (UHMR) Orbitrap instrument. 1–3 μL of buffer-exchanged AqpZ was loaded into a gold-coated glass capillary (1.2 mm O.D.) pulled to a fine tip. A voltage of 1.0 to 1.2 kV was applied to the capillary to generate an electrospray of AqpZ proteomicelles which were directed into the mass spectrometer through a transfer capillary heated to 150 °C. Following transfer into the instrument, in-source activation (150–300 eV) was applied to liberate AqpZ from the proteomicelle and/or induce dissociation of subunits from the tetrameric complex. For intact mass measurements, ions were detected in the Orbitrap at a resolving power of 12,500 (@ *m*/*z* 200). For tandem mass spectrometry (MS^2^) experiments, ions were first isolated in the quadrupole with an isolation window of 50 *m*/*z*. Fragmentation of the protein was achieved using an additional 300 eV of activation in the higher-energy collisional dissociation (HCD) cell. Fragments and dissociated monomers were then directed into the Orbitrap for detection. MS^2^ spectra were recorded at a resolving power of 200,000 (@ *m*/*z* 200).

## Results

**Figure 1:**
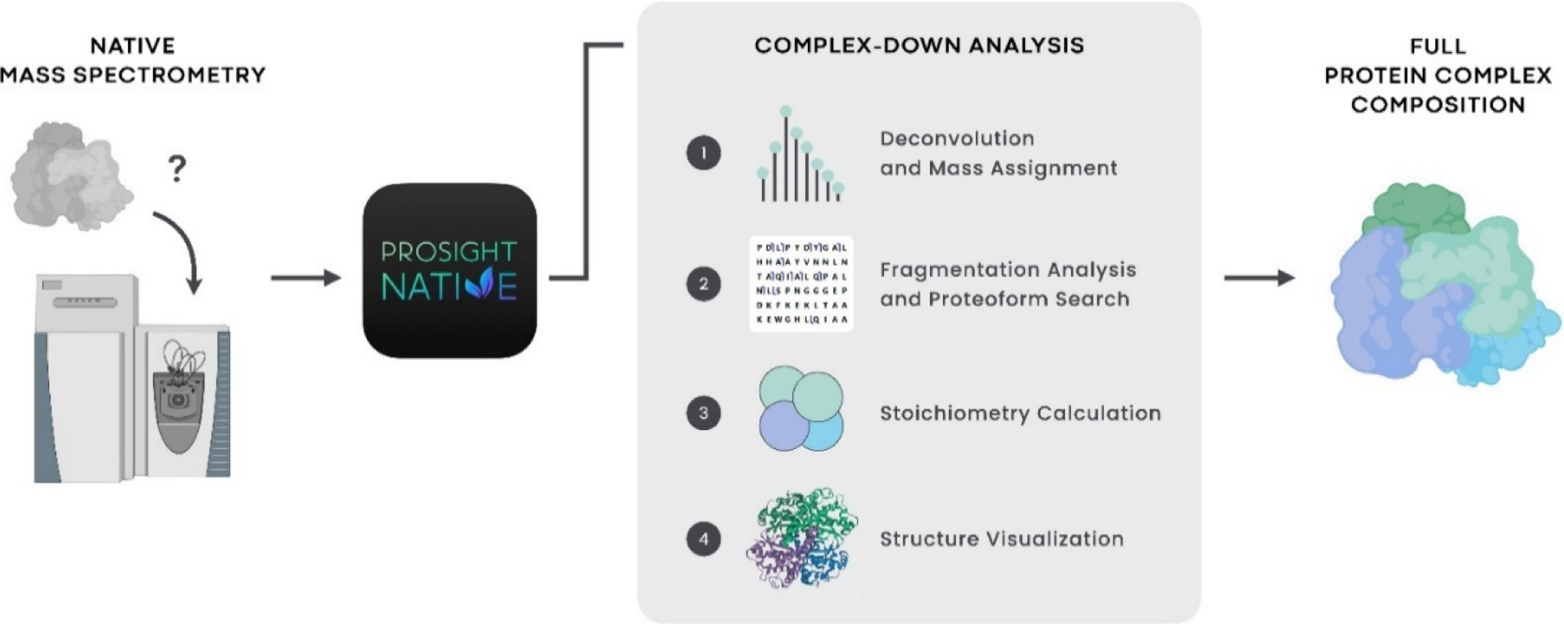
Overview of the complex-down analysis workflow in the ProSight Native software platform. (Left) Protein complexes are analyzed by mass spectrometry in native mode using the complex-down strategy, where intact complexes are measured, dissociated into monomeric subunits, and then fragmented. (Middle, 1–4) (1) The mass spectral data are then input into ProSight Native to perform mass determination of both the intact complex and the individual subunits using deconvolution. (2) Fragment ion masses are detected from fragmentation spectra and a database search is performed to obtain confident proteoform identifications of the monomers. (3) The data components are collated, and calculations determine the stoichiometry of each subunit as well as potential noncovalently bound ligands that may have been present on the intact complex. (4) Structures can be visualized along with data from the top-down experiment (e.g., residue cleavage sites) integrated directly onto the structure. (Right) The full composition of the analyzed protein complex is obtained and reported.

ProSight Native has a dedicated workflow for complex-down data analysis (Figure 1). In the following sections, we discuss the three main components of the analysis: complex mass determination, subunit identification, and stoichiometry calculation. Then, we demonstrate how ProSight Native can be used to resolve complex data. Lastly, we present software features that enable three-dimensional mapping of top-down fragmentation data onto protein structures.

### Complex Mass Determination

An integral step in analyzing intact proteins by mass spectrometry is spectral deconvolution, which converts *m*/*z* spectral peaks into masses. Having information on the molecular weight of a protein or protein complex is critical for downstream inferences such as database searches and stoichiometry calculations. For high-resolution analyses, isotopic resolution can be achieved for every charge state from masses of ∼30 kDa or smaller. However, in nMS, proteins and protein complexes analyzed are frequently >30 kDa and are therefore not typically isotopically resolved. ProSight Native has two deconvolution algorithms to handle both isotopically resolved and -unresolved data. For isotopically resolved data, ProSight Native uses a modified THRASH algorithm to obtain masses from the isotopic spacing of each charge state.^17^ For isotopically unresolved data, ProSight Native incorporates a newly modified kDecon routine that instead uses the spacing between charge states.^18,19^ The kDecon algorithm was modified to accommodate the lower number of charge states present in nMS data. Emphasis has been placed on reducing false positives, particularly from harmonic peaks that can lead to incorrect assignments (e.g., using charge states at half or twice the real values). Much of the improvement has been a result of additional logic requiring that the “shape” of a charge state distribution does not deviate more than might be expected. For instance, jagged distribution shapes are generally indicative of harmonic masses (such as 2 or 3× the actual mass) and therefore are penalized by the algorithm. ProSight Native automatically applies kDecon to lower resolution spectra at or below 15,000 resolving power. The user can also manually specify which algorithm to apply.

To perform deconvolution in ProSight Native, a user can select a spectral region of interest from the chromatogram through a graphical interface (Figure S1, top). The scans from the region are averaged together and automatically deconvoluted (Figure S1, bottom), with masses reported in a data grid and annotated on the spectrum (Figure S1, right).

Protein deconvolution is relatively straightforward for well-resolved species or masses with classic charge state distributions. However, nMS analytes, in contrast to denatured analytes, form fewer multiply charged species.^20^ At times, only two or three charge states are observable in a spectrum. These limited native charge state distributions pose a significant challenge for charge state deconvolution, increasing the likelihood of missed mass assignments and false positives due to fewer charge state confirmations of a mass. ProSight Native provides two options for determining masses in reduced charge state scenarios. First, the software has a native charge state mass determination mode that requires fewer charge states when assigning a mass. Second, users can manually match spectral peaks for masses through a graphical overlay of theoretical charge states (Figure S2). Overall, the deconvolution in ProSight Native provides mass determination of complexes that serves as the foundation for determining the complex composition.

### Subunit Identification

As with complex mass determination, ProSight Native deconvolutes subunit spectra using THRASH for isotopically resolved spectra or kDecon for unresolved spectra. Following subunit mass assignment, the software performs an intact mass tag search to identify candidate proteoforms. In addition, subunit masses are automatically linked to isotopically resolved fragmentation spectra. This step can also be manually performed, which can be helpful if data were collected in separate files or if a specific fragmentation spectrum is of interest. Once subunit fragmentation spectra are assigned, THRASH determines the masses of all fragments and a top-down database search is performed. Ion types for the search are automatically pulled from the scan header but can also be overridden by the user. The search calculates a *P*-score^21^ and a native fragmentation propensity score (nFPS) for all results.^22^ Because nMS typically yields significantly less fragmentation coverage than with denaturing conditions, the nFPS can add confidence to search results that display classic native fragmentation patterns, even in instances where relatively few fragment ions are matched.^23^

Once the search is performed, subunit identification results can be further verified. A section below provides more detail on the available validation features, including the integrated TDValidator module. In total, the subunit search of ProSight Native provides a robust set of tools for determining subunit masses and then identifying and characterizing the underlying proteoform.

### Stoichiometry Calculation

After intact complex masses and subunit proteoforms have been determined, the next step is to determine the stoichiometric ratios in which these subunits are assembled into complexes. Unlike complexes that comprise multiples of the same subunit (e.g., ligand-free homodimers), complexes with different subunits and noncovalently bound ligands present challenges in calculating stoichiometry. ProSight Native automates the process by finding complex-associated subunit combinations with the smallest mass differences between the observed and the theoretical complex mass. For each possible stoichiometry result, known protein–protein interactions for subunits in a candidate complex are downloaded from the UniProt API using the associated protein entry accessions. We query the API end point (rest.uniprot.org/uniprotkb/search) with “reviewed: true”, “accession: [accession]” and “format: XML” to retrieve reviewed protein entries in XML format. “Interactant” elements in the XML files are parsed to identify annotated protein–protein interactions for each subunit, and if both protein subunits are included in the candidate complex, the complex is annotated with the known interaction. Additionally, users can lookup stoichiometry results in CORUM and IntAct, which are databases of experimentally characterized protein complexes.^24^ These online resources leverage previous experimental evidence to weigh the stoichiometry outcomes.

**Figure 2:**
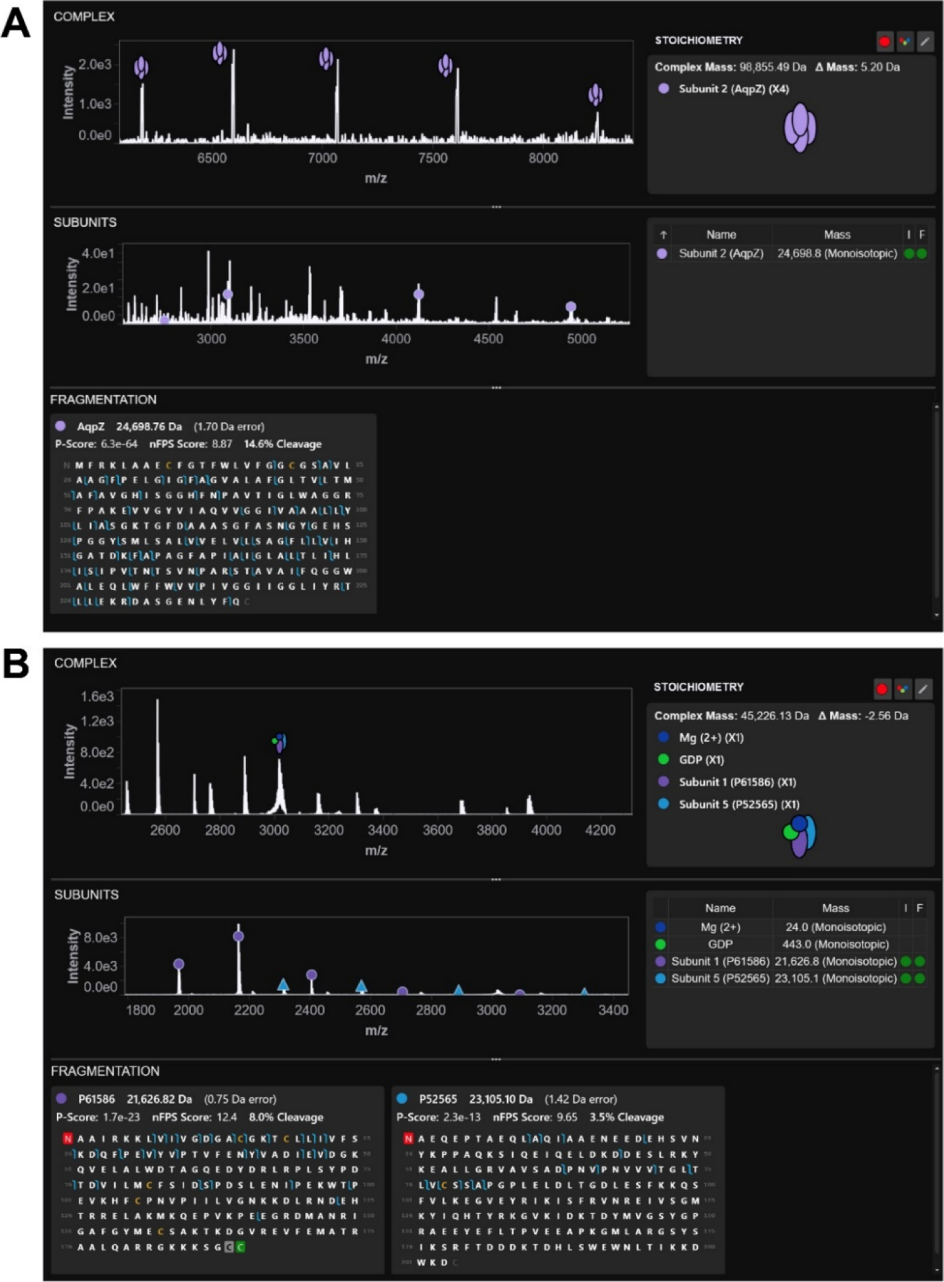
Complex composition summary from ProSight Native. (A) Aquaporin Z was analyzed by ProSight Native to determine (A, top) the complex mass, (A, middle) subunit mass, and (A, bottom) subunit identification. These data components informed the stoichiometry inference that yielded a homotetramer of full-length, unmodified Aquaporin Z. (B) A protein complex of RhoGDI1 and RhoA was analyzed by ProSight Native. As in A, the software determined (B, top) the complex mass, (B, middle) the subunit masses, and (B, bottom) the subunit identifications. Furthermore, ligands known to associate with RhoGDI1 and RhoA were included in the stoichiometry calculation. The final composition result was a heterodimer of RhoGDI1 and RhoA with noncovalently bound Mg^2+^ and GDP.

Combinations of subunit masses alone do not always account for the observed mass of an intact protein complex. Small molecule ligands can be present on the intact complex but are then lost during the subunit ejection step due to poor ionization efficiency or falling under the *m*/*z* scan range required for nMS acquisition. Considering noncovalent or labile small molecule ligands is therefore a useful strategy to account for the mass differences in the complex. We implemented a feature in ProSight Native that incorporates additional masses into the stoichiometry calculation. The candidate ligand masses can be custom masses added by the user, compounds retrieved from ChEBI, or associated cofactors imported directly from the UniProt entries associated with identified subunits. A minimum and maximum number of observable instances for each element can be set to prevent combinatorial explosion during stoichiometry calculation. For each complex mass, a final composition is assigned by the user and all supporting spectral and fragmentation data are collected into a single result output (Figure 2).

### Use Case One: Membrane Protein Complex Analysis

To illustrate the combined potential of complex-down MS and ProSight Native for structural biology applications, we analyzed nMS complex-down data collected on the *E. coli* integral membrane complex Aquaporin Z (AqpZ). Membrane proteins require stabilization of hydrophobic regions using membrane mimetics. Because detergents are still the most common way to stabilize membrane proteins, the detergent micelles need to be stripped from the protein complex to produce a clean spectrum. For this study, we used the in-source trapping (IST) available on the Q Exactive UHMR to remove the detergent surrounding AqpZ. The IST voltage was optimized at 200 eV such that AqpZ was liberated from the detergent micelles while also maintaining the noncovalent interactions of the intact complex (Figure S3A). Increasing the IST value to 250 or 300 eV increased subunit ejection and contaminant protein abundance in the spectra (Figure S3B,C).

As the first step to analyzing the AqpZ protein complex, we deconvoluted the IST 200 data using kDecon, which revealed three major species with an average mass of 98,861.9, 98,891.3, and 98,950.1 Da (Figure 2A, top). The mass differences between the species corresponded to formylation adducts.

For the experimental setup, individual charge states of the intact complex were isolated in the quadrupole and subjected to additional activation via HCD to eject and fragment the subunits. We isolated and activated several AqpZ complex charge states. The highest charge state, 16+ at *m*/*z* 6184, corresponded with the greatest degree of subunit ejection as well as the most extensive subunit fragmentation of any charge state analyzed.

The second step in complex-down data analysis is subunit mass determination and identification. Here, we used THRASH to determine the subunit masses. A main subunit form was found at 24,698.81 Da (Figure 2A, middle), along with several less abundant forms between 24,682 and 24,728 Da. THRASH also found masses in the lower *m*/*z* region of the spectrum that corresponded to fragment ions of the subunits. For identification, all THRASH masses were input for a top-down search against AqpZ, which returned a confident hit of the unmodified full-length AqpZ sequence (Figure 2A, bottom). The hit contained 89 matching fragment ions covering both the N- and C-termini. The matching ions produced extremely confident fragmentation metrics, including a *P*-score of 6.3 × 10^–64^, a highly confident nFPS of 8.87, and 29.2% of residues cleaved (Figure 2A). Furthermore, a second hit matching an N-terminally formylated full-length AqpZ sequence accounted for the majority of the remaining fragment ions in the spectrum (Figure S4).

For the third and final step of the complex-down data analysis, we calculated the stoichiometry of the complex using the mass of the complex and the subunits determined in the previous steps. The simplest theoretical composition for the 98,861.9 Da complex was a homotetramer of the unmodified AqpZ, yielding a mass of that was within ∼5 Da of the experimentally observed complex mass (Figure 2A).

### Use Case Two: Multi-Proteoform and Cofactor Complex Analysis

Complex-down experiments can also involve multiple proteins, proteoforms with modifications, and small molecule components. To demonstrate the handling of a multifaceted protein complex by ProSight Native, we analyzed previously published nMS spectra of RhoA and RhoGDI1.^25^ RhoGDI1 is a GTP-binding protein that is known to associate with RhoA.

Using ProSight Native, we deconvoluted the isotopically resolved spectrum of RhoGDI1, resulting in major species of 45,195.4, 45,272.0, and 45,296.7 Da (Figure 2B, top). Within the same spectrum, we also identified smaller mass species of 21,626.8 Da with +21.6 Da and +466.1 Da adducts and a species of 21,103.8 Da with similar +21.7 and +464.9 Da adducts.

In the existing data set, the major 21,626.8 and 23,103.8 Da species had been isolated and fragmented with HCD (Figure 2B, middle). These two fragmentation spectra were searched against the sequences of RhoGDI1 and RhoA. We first identified the 23,103.8 Da species as RhoGDI1 with N-terminal Met cleavage and an N-terminal acetyl that matched within 1.4 Da to the intact mass (Figure 2B, bottom right). While the *P*-score was a modest value of 2.3 × 10^–13^, a high nFPS of 9.65 indicated specific native fragmentation patterns not easily obtainable by decoy searches. We also observed fragmentation covering 9.9% of residues after a spectral calibration of 3.4 ppm in TDValidator. Next, we identified the 21,626.8 Da species as residues 2–189 from RhoA, derived from an N-terminal Met cleavage and an annotated C-terminal propeptide. The proteoform was also found to be modified via N-terminal acetylation, C-terminal methylation, and S-geranylgeranylation at the C-terminal cysteine with less than 1 Da intact mass error (Figure 2B, bottom left). We observed a confident *P*-score of 1.7 × 10^–23^, a confident nFPS score of 12.4, and matching fragments covering 24% of residues following TDValidator analysis.

Using the stoichiometry inference tool within ProSight Native, the best stoichiometry result was a heterodimer of RhoA and RhoGDI1 (−464.8 Da error), which was automatically highlighted in the software as a Uniprot-annotated protein–protein interaction. When we included known cofactors and other small molecules previously associated with RhoA and RhoGDI1, the mass difference was reduced to −1.2 Da. The final composition was RhoA-RhoGDI1-GDP-Mg^2+^. We additionally identified minor mass species within the MS^1^ spectrum corresponding to the GTP-bound form of the complex (−0.7 Da error) as well as minor monomer forms such as phosphorylated RhoA (21,706.1 Da observed, 0.1 Da error), RhoA bound to Mg^2+^ and GDP (22,092.9 Da observed, 0.2 Da error), and RhoGDI1 bound to Mg^2+^ and GDP (23,568.7 Da observed, 2.0 Da error).

### Validating Native Proteoforms

We integrated TDValidator for users to validate fragment ions through an interactive combination of fragment maps and spectral annotation. TDValidator relies on an isotopic distribution fitting algorithm to match theoretical fragment ions derived from the chemical formula of the proteoform-of-interest rather than the averagine distribution used in THRASH. In contrast to THRASH mass discovery, the isotopic fitting approach can increase sequence coverage by looking for specific ions. Adding several more matching ions can substantially improve confidence metrics of native fragmentation spectra with a few matching fragment ions. Conversely, fragment ions that poorly match their theoretical isotope distributions can be quickly identified and excluded from results.

Several tools within TDValidator can extend the depth of fragmentation analysis, which can be particularly important in cases with a limited number of fragment ions. First, users can overlay the theoretical isotope distribution on top of the experimental fragment ions. Second, proteoforms can be edited with custom masses and modifications such that different proteoforms, including proteoforms not identified during search time, can be compared against the same spectrum. Third, to more accurately define isotopes that contain metal ions commonly seen in nMS, users can generate isotopic distributions with the BRAIN algorithm.^26^ Additionally, TDValidator allows users to match internal fragment ions, determine calibration shifts, and measure false positive likelihoods through decoy proteoform matching.

To customize the TDValidator module for ProSight Native, we considered the differences in fragmentation behavior between native and denatured top-down fragmentation. Because native proteoforms are retained in their compact native state, fewer fragment ions are formed compared with when the same proteins are denatured and unfolded. Despite reduced backbone cleavages, proteins are more likely to fragment along certain fragmentation channels in nMS, creating sequence “hotspots”.^22^ For instance, phenylalanine–tryptophan pairs fragment at rates several-fold higher than under denaturing conditions. Similarly, while residues C-terminal to aspartic acid and N-terminal to proline fragment readily in denatured proteins, they fragment at even higher rates in native proteins. To highlight the differences in fragmentation, TDValidator automatically color-codes native fragmentation hotspots in the fragment map and spectra.

**Figure 3:**
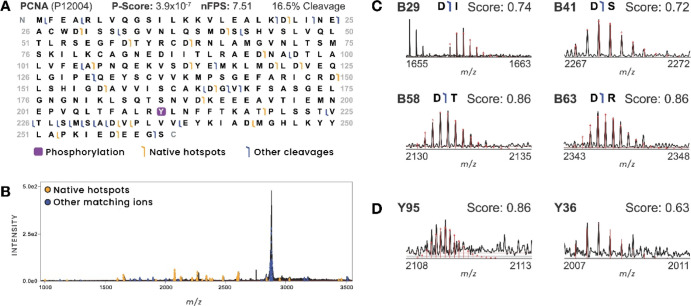
Validating native fragmentation spectra using ProSight Native. (A) Matching fragment ions are mapped onto a PCNA proteoform sequence. (B) The TDValidator module within ProSight Native annotates fragmentation spectra with matching fragment ions. Users can interrogate the isotope distributions for fragments central to the proteoform spectral match. Confirming fragment ions at native fragmentation hotspots are colored in yellow. Other matching fragment ions are shown in blue. (C) Well-fit fragment ions shown correspond to cleavages at sites C-terminal to an aspartic acid residue, which is a dominant cleavage site in native MS. (D) Confirming cleavage sites flanking modifications (shown here for phospho-Tyr211) that improved confidence in the phosphorylation localization are shown. Scores shown are isotope fit scores from TDValidator. Fragment ions with scores greater than 0.50 were used.

Next, we sought to demonstrate the fragmentation tools in ProSight Native on existing spectra from human proliferating cell nuclear antigen (PCNA). Following mass determination (Figure S5) and top-down search, we identified a proteoform of UniProtP12004phosphorylated at Tyr211 (−16.3 Da intact error) with a *P*-score of 4.2 × 10^–6^ and 6% of residues cleaved (15 matching fragments). Using the TDValidator module, this sequence coverage was increased to 16.5% of residues cleaved, with 30 additional matching fragments. We also examined fragmentation patterns and found 20 fragment ions at native hotspots, leading to a confident nFPS of 7.51. We then visualized these results on a fragment map (Figure 3A, orange flags) and on the spectrum (Figure 3B, orange ions). Further interrogation of hotspot ions revealed their theoretical and experimental isotopic distributions to be well-matched (Figure 3C), with isotopic fit scores >0.7. In general, we use 0.5 as the lower limit for isotopic fit scores and manually validate ions with fit scores between 0.5 and 0.7 to ensure the theoretical distributions are indeed well fit to the data. Lastly, we localized a phosphorylation modification at Tyr211, a previously annotated phosphosite (Figure 3D).^27^

### Visualizing Native Proteoform Structures

To streamline the process of going from MS data to structural biology insights, we next developed the Structural Viewer in ProSight Native to superimpose top-down data onto three-dimensional proteoform structures. Top-down-informed structures can be used to validate the localizations of PTMs from top-down search results and to visualize these PTMs relative to enzyme active sites, binding pockets, and other structural features. Additionally, mapping fragmentation data onto structures can help determine solvent accessibility across proteoform regions as accessibility is typically proportional to fragmentation levels. Likewise, these results can shed light on a proteoform’s fragmentation efficiency, for example, why a proteoform region exhibits poor fragmentation.

Structural Viewer takes the accession numbers of resultant proteoforms from a top-down search and retrieves their corresponding structures from UniProt, RCSB, and AlphaFold.^28^ For each structure, the software displays monomers that comprise the structure and indicates whether any monomers match the sequence of the resultant proteoform. The selected structure is displayed in an interactive viewer powered by the JavaScript library NGL Viewer.^29^ Here, fragment ions and PTMs of the proteoform fragmentation results can be added to the structure. We envision a wide range of potential use cases for the Structural Viewer, including mapping extensive top-down fragmentation,^30^ pairing top-down fragmentation with cross-linking data, and visualizing other structural information such as *B*-factor to better understand protein complex structures.^31^

**Figure 4:**
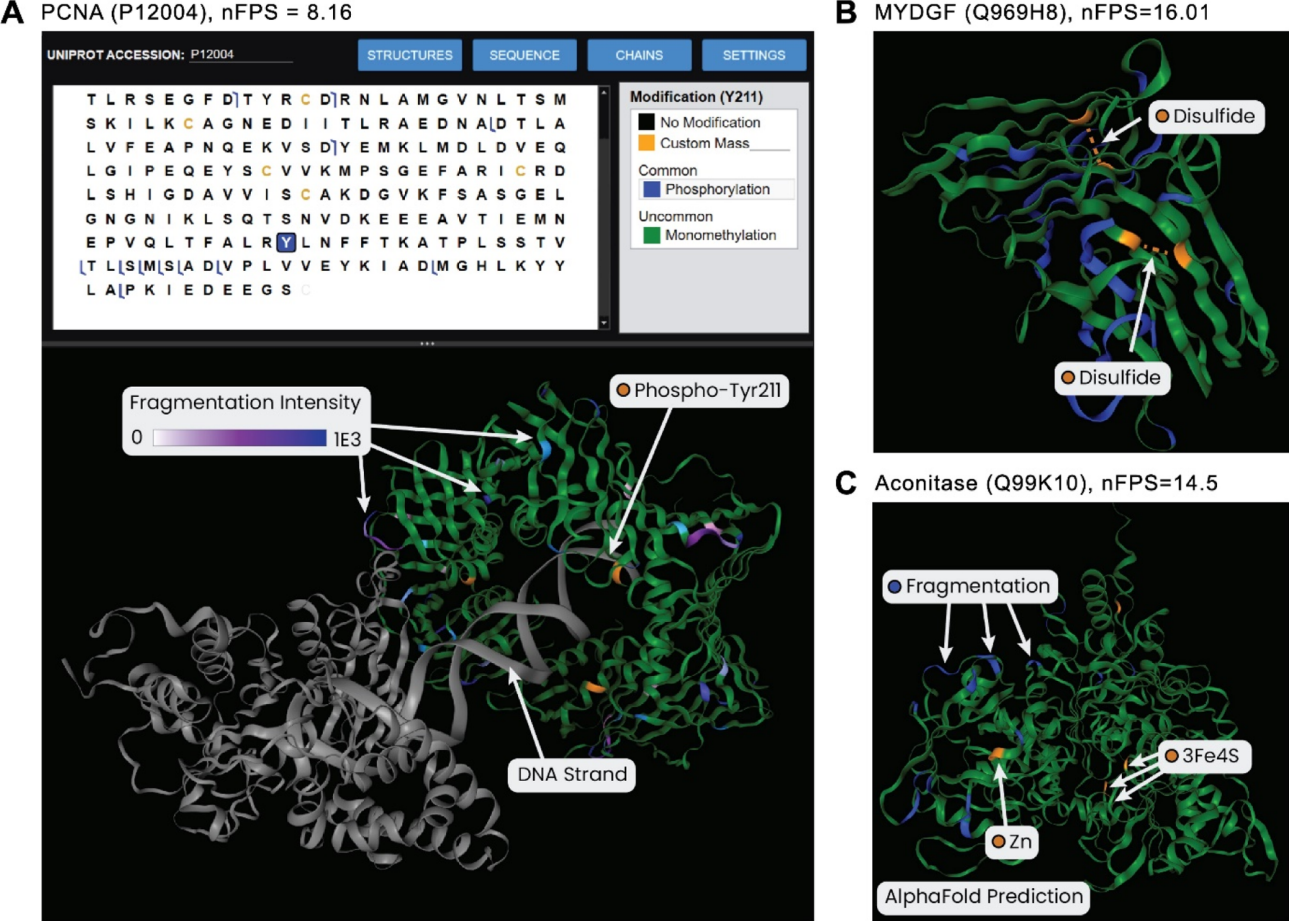
Structural viewing module for relating complex-down and native MS experiments to three-dimensional structures. (A) The Structural Viewing module allows users to choose experimental structures available from RCSB, custom PDB files, or predicted structures from AlphaFold. Detected fragments and PTMs can be mapped onto the 3D structure to aid with data validation and generating structural biology insights. In the structure shown, PCNA is observed as a homotrimeric ring structure with a central DNA-binding cavity. Fragments are observed only at the exterior of the structure, and the top-down localized phospho-Tyr211 is within the central cavity in close proximity to the bound DNA. (B) A homodimer of MYDGF is shown. The detected MYDGF proteoform includes a disulfide linkage, which is consistent with the proximity of these two cysteines. (C) While murine aconitase does not yet contain an experimental structure, the proteoform can be mapped onto the AlphaFold prediction in ProSight Native. Here, fragmentation was observed only at the exterior of the predicted structure, mainly at highly probable sites for native fragmentation. This observation is consistent with the typical fragmentation of large globular proteins in their native state, as residues on the exterior of the protein near the termini are preferentially cleaved. For all structures, green denotes a relevant monomer, blue marks a top-down cleavage site, and yellow represents a modification from the proteoform-of-interest.

We next brought the PCNA data from Figure 3 into the Structural Viewer. The structural entry (PDB 7NVO) shows a homotrimeric PCNA complex that forms a ring-like structure around a DNA strand. We found that the PCNA structure corroborates the top-down fragmentation patterns from the search result; matching fragments are observed at the exterior of the ring, suggesting that this native structure was retained in the gas phase (Figure 4A). The phosphorylated Tyr211 can also be seen near the DNA-binding cavity of the complex, a site previously shown to be integral to chromatin-bound PCNA stability.^32^

As a second example, we analyzed existing native spectra of myeloid-derived growth factor (MYDGF, UniProt Q969H8). A search revealed the proteoform as the full-length chain expected after cleavage of an annotated signal sequence (Figure S6). The identified proteoform includes a disulfide bond between the two cysteine residues in the sequence (Cys63 and Cys92). When the proteoform was mapped onto its homodimeric structure (PDB 6SVK), we observed that these two cysteines of each monomer were close in space, consistent with the formation of a disulfide linkage.

While experimental structures are available for many proteins, we wanted to extend structure viewing to those without published structures. We thus integrated calls to the AlphaFold API within ProSight Native, which gives users access to predicted structures for most entries in UniProt. Next, we analyzed an existing native top-down data set of murine aconitase that currently has no structure entry in RCSB. In ProSight Native, we detected an intact mass of 82,868.6 Da via kDecon (Figure S7). Top-down search identified a proteoform from UniProt accession Q99KI0 with a cleaved N-terminal transit peptide (residues 1–27) and an N-terminal pyroglutamate. This proteoform identification had an intact mass difference of 421.2 Da, which is consistent with a 3Fe4S cluster and two Zn^2+^ ions (−1.8 Da error), two cofactors previously shown to bind aconitase. Using the TDValidator module within ProSight Native, one Zn^2+^ binding site could be localized to Asp687, but the 3Fe4S cluster and remaining Zn^2+^ ion could not be localized. We then mapped an aconitase proteoform containing localized Zn^2+^ and a previously annotated binding site for 3Fe4S (cysteines 385, 448, and 451) to its predicted AlphaFold structure. We observed the matching fragments to be at the exterior of the structure, which was an expected result due to the compact state of native proteins. The structure also showed that the annotated 3Fe4S binding cysteine residues were located in the interior of the structure. This interior location was also unsurprising, given the lack of fragmentation coverage for 3Fe4S. Overall, our examples of top-down informed structures highlight the research potential of unifying nMS data with three-dimensional structures.

## Conclusions

nMS is a powerful tool for analyzing protein assemblies. However, large-scale adoption has been hindered to date by the lack of cohesive and complete nMS software offerings. Introduced here, ProSight Native is a software platform that can take a user from the start to the finish of nMS protein complex data analysis. ProSight Native provides a host of analysis features with the goal of making native protein and complex-down mass spectrometry an easier and more attractive experimental option in future structural biology studies.

## Data Availability

Aquaporin data have been deposited to the ProteomeXchange Consortium via the PRIDE partner repository with the data set identifier PXD038817. Other data used in this article are available as described in the original Skinner et al. publication.^25^

## Supporting Information Available

The following Supporting Information is available free of charge at ACS Web site. The Supporting Information is available free of charge at https://pubs.acs.org/doi/10.1021/acs.jproteome.3c00171.

Intact protein analysis in ProSight Native, manual mass determination, effect of IST on Aquaporin Z, annotated fragmentation spectrum of Aquaporin Z, native MS analysis of PCNA, native MS analysis of MYGDF, and native MS analysis of aconitase (PDF)

## Notes

Software Availability: ProSight Native is available for download from the Proteinaceous web site at proteinaceous.net/prosightnative. Free 60 day license keys will be provided upon request.
