# Supplementary material for: ProSight Native: Defining Protein Complex Composition from Native Top-Down Mass Spectrometry Data
Source: J Proteome Res. 2023 Jul 12;22(8):2660–8. doi: 10.1021/acs.jproteome.3c00171 (PMC10407923; doi:10.1021/acs.jproteome.3c00171)
Supplement: Supplementary file 1 — pr3c00171_si_001.pdf [file pr3c00171_si_001.pdf]

Supporting Information

ProSight Native: Defining Protein Complex Composition from Native Top-Down Mass Spectrometry Data

Kenneth R. Durbin[1], Matthew T. Robey[1], Lilien N. Voong[1], Ryan T. Fellers[1,2], Corinne A. Lutomski[3,4], Tarick J. El-Baba[3,4], Carol V. Robinson[3,4], Neil L. Kelleher[1,2]*

1 Proteinaceous, Inc., Evanston, Illinois 60201, United States

2 Northwestern University, Evanston, Illinois 60208, United States

3 Department of Chemistry, University of Oxford, 12 Mansfield Rd. Oxford, UK OX1 3TA

4 Kavli Institute for NanoScience Discovery, Dorothy Crowfoot Hodgkin Building University of Oxford, Oxford UK OX1 3QU

**Corresponding Author*

Kenneth R. Durbin – kdurbin@proteinaceous.net

Table of Contents for Supporting Information

Figure S1. Intact protein analysis in ProSight Native.

Figure S2. Manual mass determination.

Figure S3. Effect of in-source trapping on Aquaporin Z.

Figure S4. Annotated fragmentation spectrum of Aquaporin Z.

Figure S5. Native MS analysis of PCNA.

Figure S6. Native MS analysis of MYGDF.

Figure S7. Native MS analysis of aconitase.

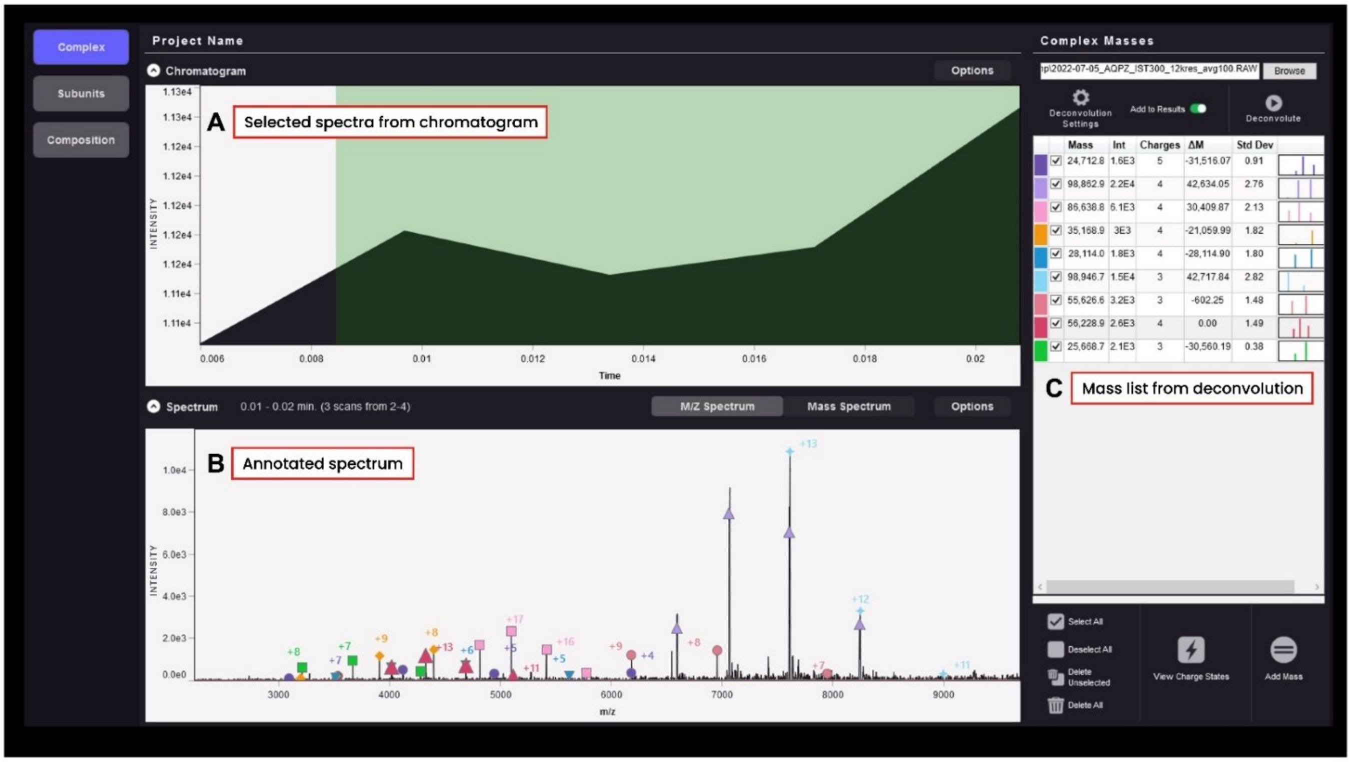

Figure S1 – Intact protein analysis in ProSight Native. (A) Spectra are selected by the user from the chromatogram. These spectra are automatically averaged together and shown in the bottom portion of the interface. (B) The averaged spectrum is deconvoluted and any masses found are annotated on the spectrum. (C) The software automatically applies kDecon for lower resolution spectra at or below 15,000 resolving power. The user can also manually specify which algorithm to apply.

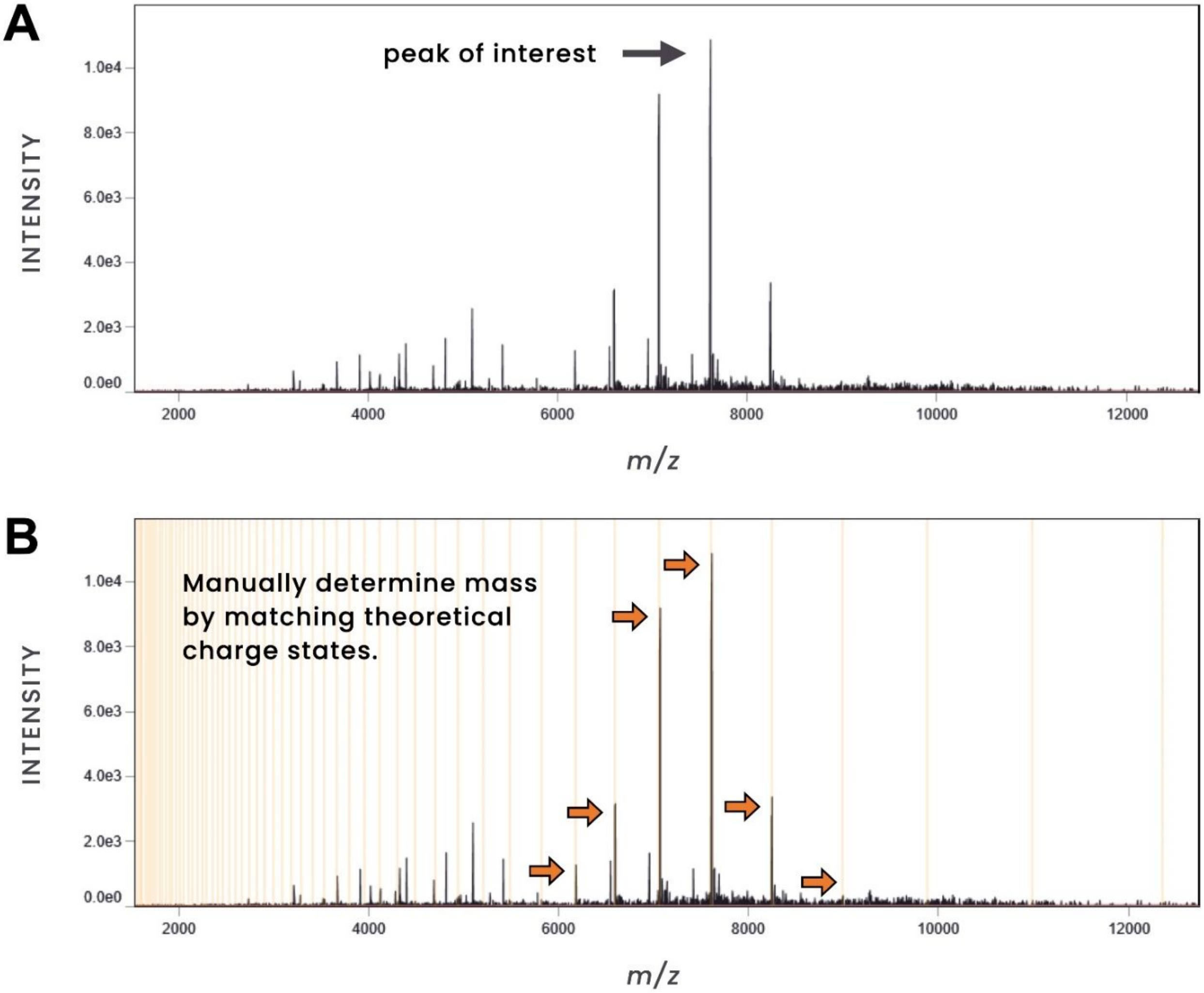

Figure S2 – Manual mass determination. (A) ProSight Native provides a feature to manually determine masses for peaks of interest. (B) The theoretical charge state distribution of a mass can be overlaid onto a spectrum. From there, a user can determine if multiple peaks from the spectrum match theoretical charge states. Peaks matching charge states from a mass of 98,813 Da are indicated with orange arrows.

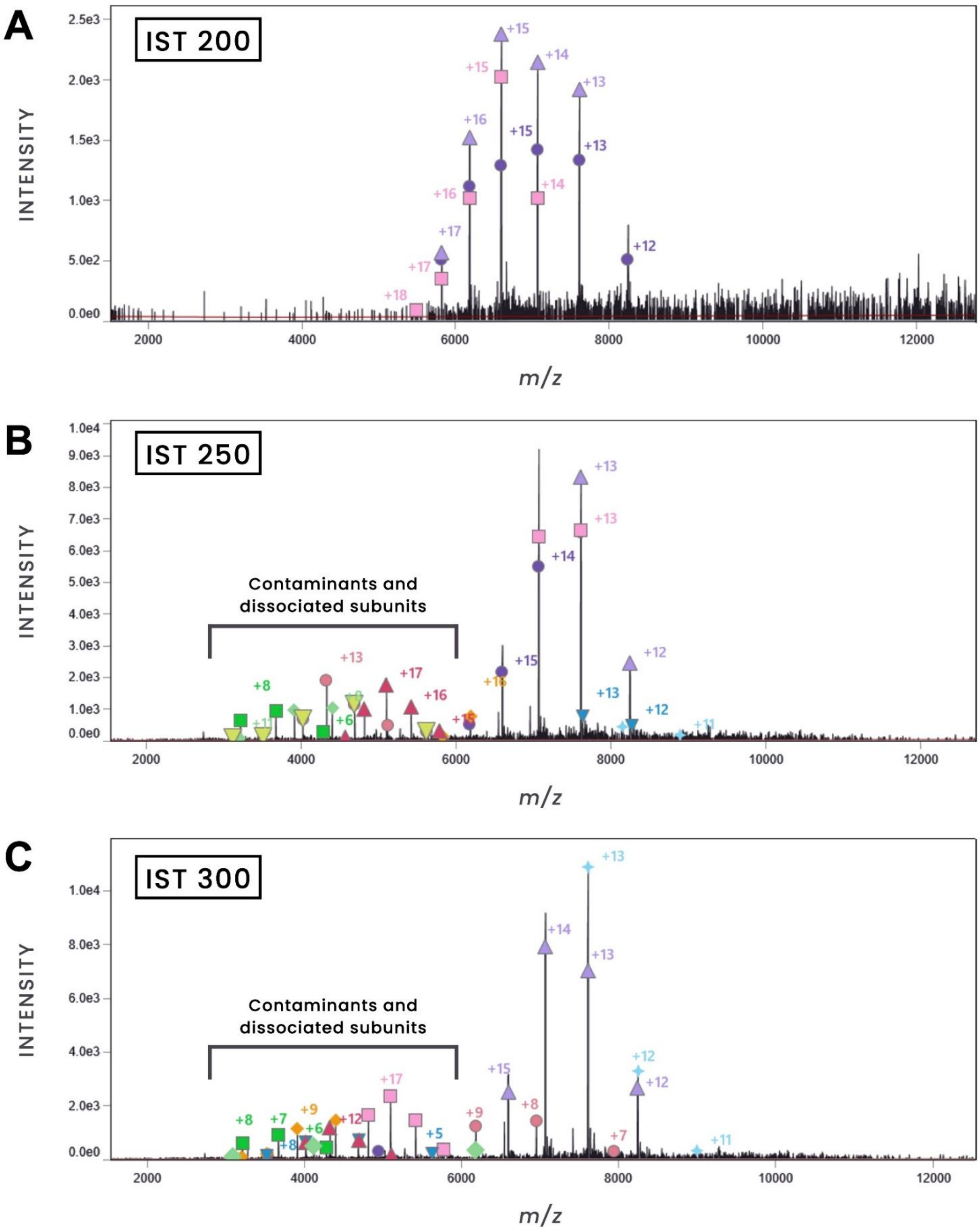

Figure S3 – Effect of in-source trapping (IST) on the intact AqpZ protein complex. Different IST levels were used to analyze ApqZ, from (A) 200 eV to (B) 250 eV to (C) 300 eV. Charge state distributions from kDecon mass determinations are annotated on each spectrum.

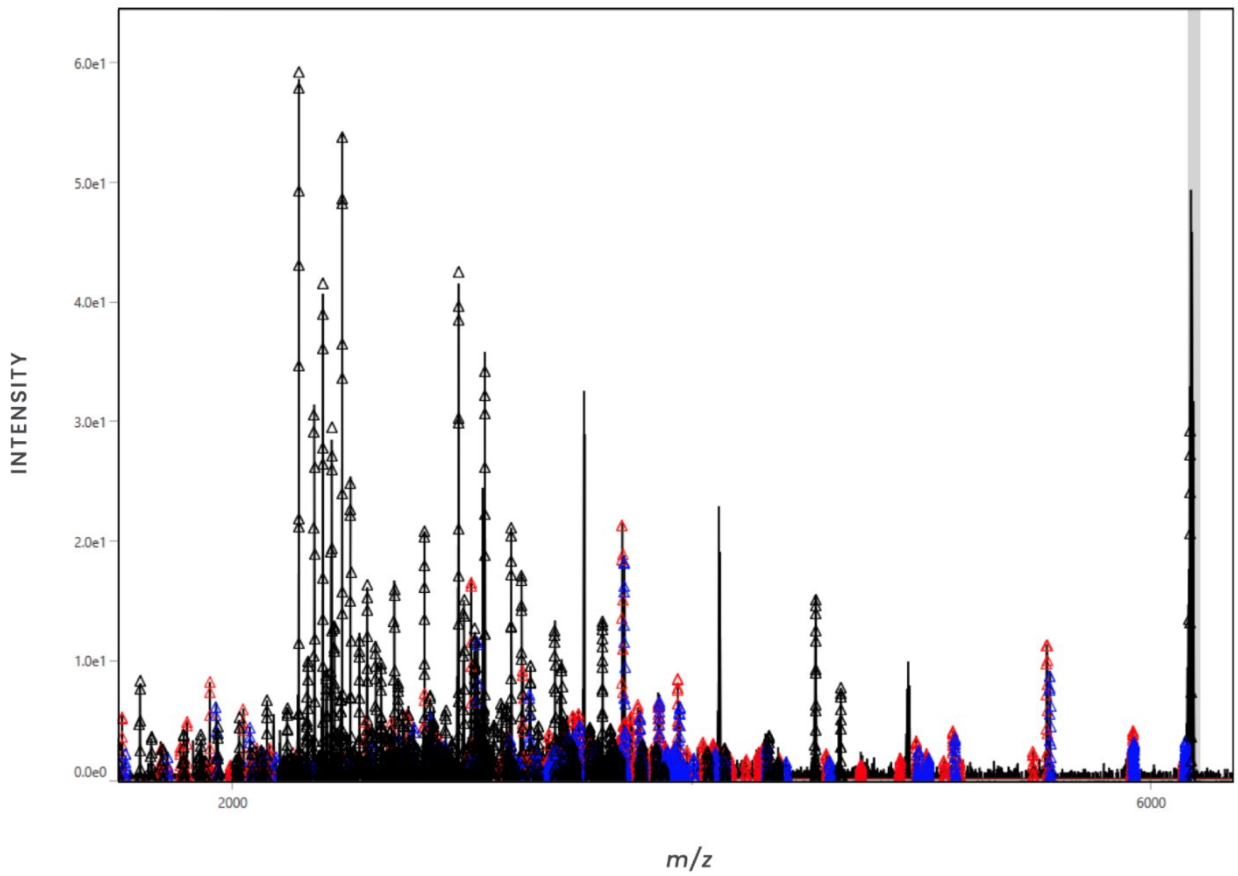

Figure S4 – Annotated fragmentation spectrum of Aquaporin Z data. Matching fragment ions are annotated on the spectrum for shared fragment ions (black), unmodified Aquaporin Z (red), and N-terminally formylated Aquaporin Z (blue). On the right side of the spectrum, the isolation window is highlighted (gray).

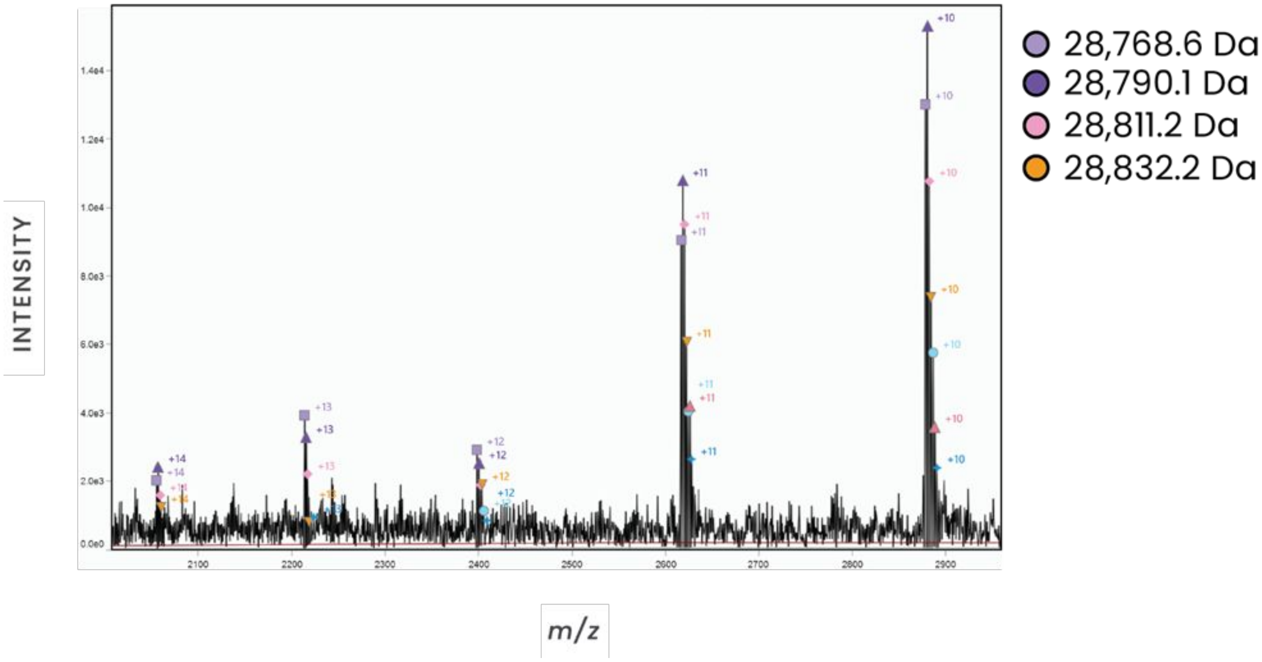

Figure S5 – Native mass spectrometry analysis of proliferating cell nuclear antigen (PCNA).

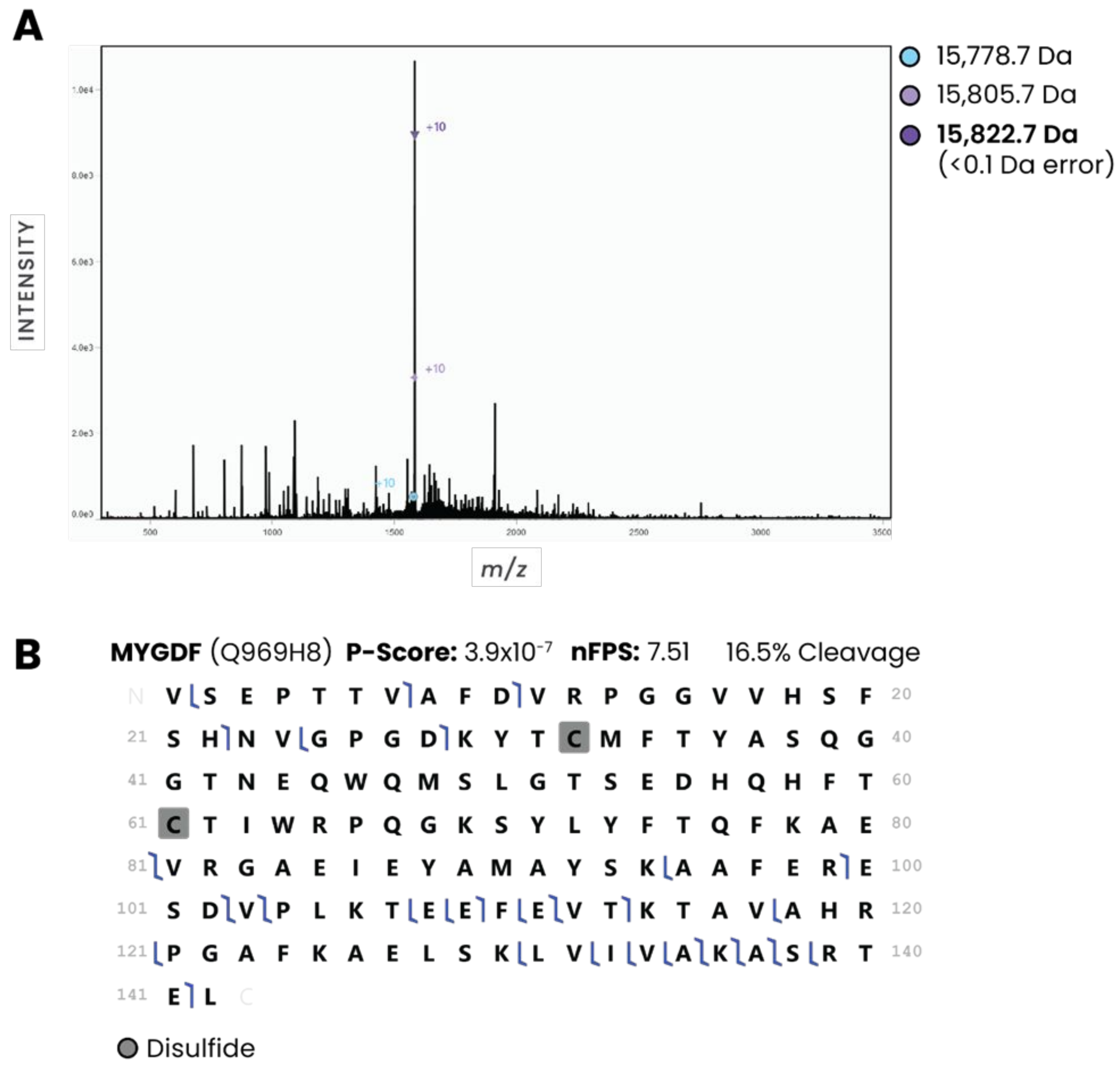

Figure S6 – Native mass spectrometry analysis of MYGDF. (A) THRASH was used in ProSight Native to deconvolve the intact spectrum of MYGDF, resulting in a major mass species of 15,822.7 Da. (B) MYGDF was confidently identified as a disulfide-modified form using a fragmentation search in ProSight Native.

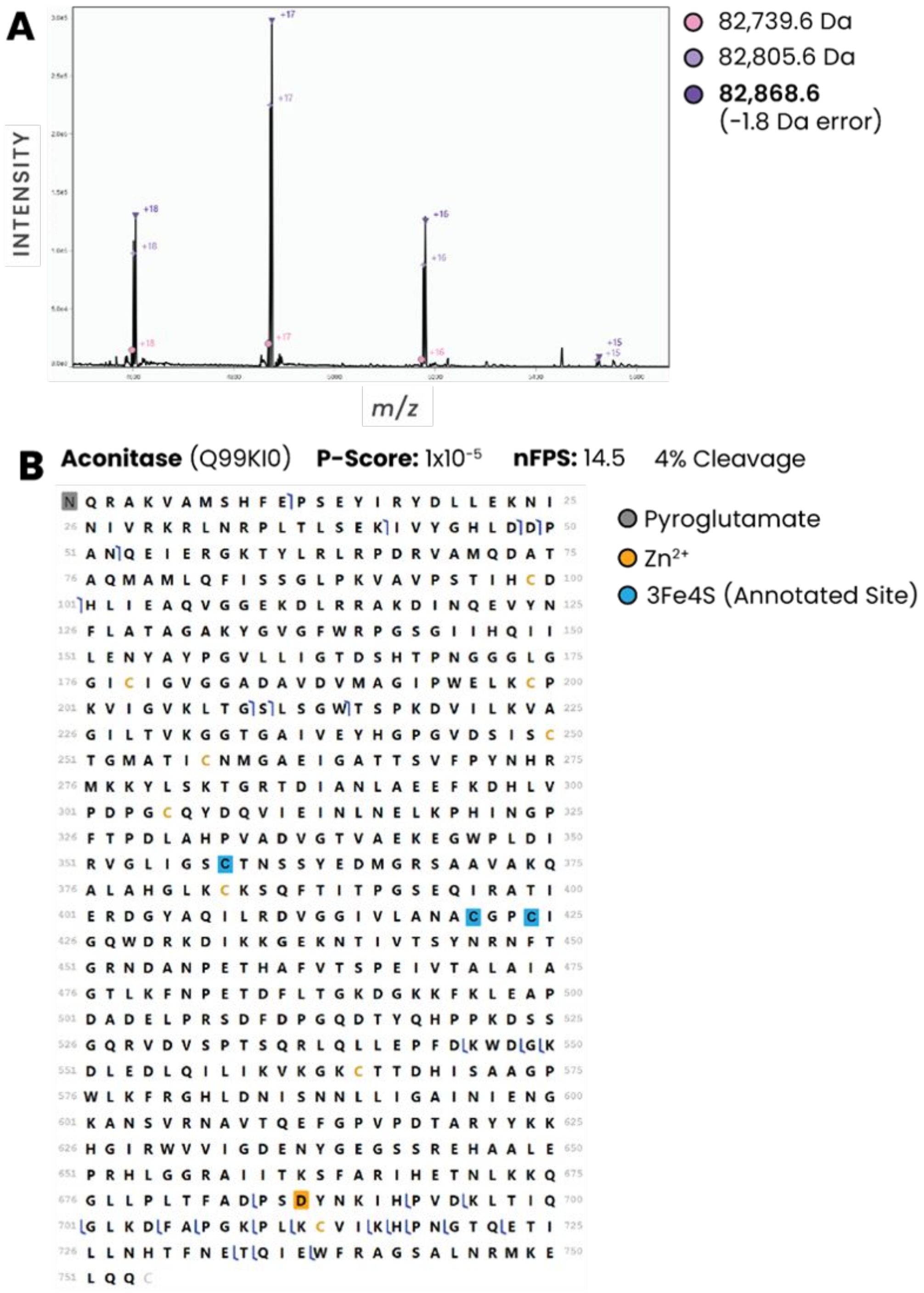

Figure S7 – Native mass spectrometry analysis of murine aconitase. (A) kDecon was used in ProSight Native to deconvolve the intact spectrum of aconitase, resulting in a major mass species of 82,868.6 Da. (B) A fragmentation search within ProSight Native confidently identified this mass as aconitase, with an N-terminal pyroglutamate and a bound Zn^{2+} cofactor. The detected intact mass is consistent with an additional 3Fe4S cofactor, with predicted cysteine binding sites highlighted on the fragment map.
